# The association between ferritin levels and all-cause mortality in stroke patients

**DOI:** 10.3389/fneur.2024.1386408

**Published:** 2024-06-26

**Authors:** Xuefen Xia, Jiongjiong Liu, Wenqiang Fang, Zhibo Chen, Jie Wang, Huiqin Xu

**Affiliations:** ^1^Department of Neurology, The First Affiliated Hospital of Wenzhou Medical University, Wenzhou, China; ^2^Department of General Practice, Fuyang Hospital Affiliated to Anhui Medical University, Anhui, China; ^3^Department of Endocrinology, The Second Affiliated Hospital of Wenzhou Medical University, Wenzhou, China

**Keywords:** ferritin, all-cause mortality, stroke, mimic, mortality, risk

## Abstract

**Purpose:**

The purpose of study was to describe the association between ferritin and all-cause mortality of cases with stroke.

**Methods:**

Clinical data derived from Multiparameter Intelligent Monitoring in Intensive Care were analyzed. The primary endpoint was 30-day mortality. The potential prognostic roles of Ferritin L were analyzed by Cox proportional hazard models. The independent prognostic roles of Ferritin L in the cases were analyzed by smooth curve fitting.

**Results:**

Concerning 30-day mortality, the HR (95% CI) for a high Ferritin (≥373) was 1.925 (1.298, 2.854; *p* = 0.00113), compared to a low ferritin (< 373). After adjusting for multiple confounders, the HR (95% CI) for a high Ferritin (≥373) was 1.782 (1.126, 2.820; *p* = 0.01367), compared to a low Ferritin (< 373). A non-linear association between Ferritin and 30-day mortality was found. Using recursive algorithm and two-piecewise linear regression model, inflection point (IP) was calculated, which was 2,204. On the left side of the IP, there was a positive relationship between Ferritin and 30-day mortality, and the effect size, 95% CI and *p* value were 1.0006 (1.0004, 1.0009) *p* < 0.0001, respectively. On the right of the IP, the effect size, 95% CI and *p* value were 1.0000 (1.0000, 1.0000) and 0.3107, respectively.

**Conclusion:**

Ferritin was associated with increased risk of stroke; it is important to further examine the association if the increased uric acid would increase the outcome of stroke in a longitudinal study. The non-linear relationship between Ferritin and all-cause mortality of stroke was observed. Ferritin was a risk factor for the outcome of stroke when ferritin was <2204.

## 1 Introduction

Stroke, a neurological deficit originating from cerebrovascular causes, disrupts blood supply to part of the brain, potentially inducing irreversible damage to deprived neural tissues and consequently triggering substantial morbidity and mortality worldwide (1–3). With various subtypes, including ischemic and hemorrhagic strokes, each presenting unique pathophysiology, risk factors, and outcomes, stroke management demands rigorous attention to detail, a comprehension of its multifaceted nature, and often, a patient-centric approach to care (4). While acute phase interventions, both medical and/or surgical, followed by rehabilitative strategies, remain fundamental to stroke management, a prudent understanding and investigation of potential prognostic markers are pivotal to interpret, forecast, and possibly enhance patient outcomes (3–5).

Ferritin, a cellular protein that binds and sequesters iron, is traditionally recognized as an indicator of iron stores within the body (6, 7). Elevated serum ferritin levels, aside from implications in iron metabolism disorders, have also been acknowledged as an acute-phase reactant, often elevating in situations of systemic inflammation, cellular injury, and specific disease states (8). Within cardiovascular diseases, elevated ferritin levels have been associated with adverse outcomes, highlighting its potential utility as a prognostic marker (9). Furthermore, emerging data insinuate that ferritin, through its involvement in iron metabolism and its capacity to induce oxidative stress, might participate in the pathophysiological cascade following ischemic cell death and in propagating secondary injury mechanisms post-stroke. Nevertheless, the explicit association between ferritin levels and outcomes in stroke patients warrants further elucidation. While stroke instigates a complex interplay of inflammatory, oxidative, and neurodegenerative processes, defining the role of ferritin—both as a marker and a participant in this cascade—requires meticulous investigation (10, 11). Given the pathophysiological complexity and heterogeneity among stroke patients, it is imperative to discern whether ferritin levels maintain a consistent, independent association with outcomes or simply reflect the inflammatory and oxidative processes that prevail following cerebral ischemia or hemorrhage (12, 13).

This study commences an exploration into the association between serum ferritin levels and all-cause mortality in stroke patients, delving into the complexities of stroke subtypes and coexisting variables that could potentially influence this relationship. By unraveling these associations, we aim to enhance our understanding of stroke pathophysiology, potentially facilitating the development of novel prognostic and therapeutic strategies aimed at mitigating the profound impact of this formidable neurological challenge. In this study, we utilized real-world data obtained from the latest Multiparameter Intelligent Monitoring in Intensive Care (MIMIC) database to assess the association between ferritin and stroke patient outcomes, adjusting for various potential confounders.

## 2 Methods

### 2.1 Study population

The present study strictly adheres to the Strengthening the Reporting of Observational Studies in Epidemiology (STROBE) statement to ensure rigorous and transparent reporting (14). Data were meticulously extracted from the Medical Information Mart for Intensive Care IV (MIMIC-IV) database, version 2.2 (15, 16), which encompasses a comprehensive array of variables, including vital signs, medications, and demographic information, from patients admitted to the Intensive Care Unit (ICU) at the Beth Israel Deaconess Medical Center (BIDMC, Boston) from 2008 to 2019, totaling 76,943 distinct admissions. Ethical approval was diligently secured from the Massachusetts Institute of Technology (MIT) and the Institutional Review Boards prior to data utilization, and all researchers completed the “Data or Specimens Only Research” online course, ensuring adherence to ethical and practical guidelines for data handling.

Inclusion criteria were strictly delineated as patients aged over 16 years who were initially admitted to the hospital for stroke and had a hospitalization duration exceeding 1 day. Patients were excluded based on the following criteria: (a) presence of acute infection, (b) diagnosis of malignancy, (c) critical hepatic illness, indicated by aspartate transaminase or alanine transaminase levels exceeding five times the upper normal limit, and (d) kidney diseases, manifested by an estimated glomerular filtration rate below 30 mL/min or necessitating dialysis due to end-stage renal failure.

### 2.2 Study variables and outcomes

Patient demographic data, vital signs, laboratory characteristics, comorbidities, and scoring systems were systematically compiled and analyzed. Vital signs monitored within the initial 24 h post-ICU admission encompassed heart rate, oxygen saturation (SpO2), systolic blood pressure (SBP), and diastolic blood pressure (DBP). A comprehensive list of comorbidities, inclusive but not restricted to coronary heart disease (CHD) and chronic heart failure (CHF), was assembled. Laboratory measurements, such as the white blood cell (WBC) count within the first 24 h, and Glasgow Coma Scale (GCS) scores, were also incorporated to provide a comprehensive clinical picture.

The primary outcome was meticulously defined as the 30-day mortality rate, with secondary outcomes extending to 90-day and 1-year mortality rates. The commencement of the follow-up was marked from the participants’ admission date, ensuring a minimum follow-up duration of 1 year for all participants. Death dates were accurately procured from Social Security Death Index records, ensuring reliable outcome data.

### 2.3 Statistical analysis

Participants were categorized into three groups according to their ferritin levels. Continuous data were expressed as mean ± standard deviation (SD), whereas categorical data were presented as percentages or frequencies, as suitable. The Kruskal-Wallis H test and χ^2^ tests were applied to discern differences in baseline characteristics between the ferritin groups for continuous and categorical variables, respectively. Following this, Cox regression analysis was employed to ascertain the relationship between ferritin levels and stroke outcomes. In the preliminary Model 1, no adjustments were made for covariates. Model 2 incorporated adjustments for race, gender, and age. Additionally, Model 3 adjusted for confounding factors including gender, age, heart rate, SpO2, respiratory rate, hemoglobin, and chronic medical conditions such as hypertension, chronic heart failure, coronary heart disease, and the Glasgow Coma Scale score. To provide a more detailed perspective, subgroup analyses were conducted to delve deeper into the association of serum ferritin levels with 30-day mortality across distinct subgroups, offering insights into its influence across various patient demographics and medical conditions. For a structured analysis, continuous variables were dichotomized based on their clinical relevance. To address potential confounding and to reduce the impact of selection bias in estimating the effect of serum ferritin levels on stroke outcomes, a propensity score matching (PSM) analysis was implemented.

All statistical evaluations were conducted using the R software (Version 3.6.1). *p* values were considered two-sided, with a *p* value <0.05 signifying statistical significance.

## 3 Results

### 3.1 Subject characteristics

In our study, 483 stroke cases were included. The baseline demographic, laboratory and clinical features of the participants are listed in Table 1. The eligible participants included 213 women and 270 men with a mean age of 64.05 ± 16.20 years, and the mean ferritin was 1076.9 ± 4058.4. It was demonstrated that the proportions of cases with SOFA or OASIS score, 90-day mortality, 30-day mortality, and 1-year mortality were increased in the high ferritin group. The SBP and SpO_2_ were decreased (*p* < 0.05). Moreover, organ function was also assessed in the two groups using the laboratory indices, and the results showed that renal function of patients in the high ferritin group was worse than that in the high ferritin group.

**Table 1:** Baseline characteristics of the study population.

*Characteristics*	Ferritin		*p* value
	<373	≥373	
Number of patients	241	242	
Age, years	65.49 ± 16.49	62.63 ± 15.82	0.056
Sex, *n* (%)			<0.001
Male	110 (45.64)	160 (66.12)	
Female	131 (54.36)	82 (33.88)	
Ethnicity, *n* (%)			0.832
White	149 (61.83)	140 (57.85)	
Black	24 (9.96)	21 (8.68)	
Other	68 (28.22)	81 (33.47)	
Vital signs			
SBP, mmHg	126.39 ± 18.51	121.95 ± 17.82	0.005
DBP, mmHg	63.87 ± 12.93	63.88 ± 12.32	0.859
MAP, mmHg	81.20 ± 12.80	79.59 ± 12.31	0.119
Heart rate, beats/min	82.25 ± 14.43	90.50 ± 15.47	<0.001
Respiratory rate, t/min	19.20 ± 4.08	20.63 ± 4.42	<0.001
Temperature, °C	36.90 ± 0.49	36.98 ± 0.74	0.257
SpO2, %	97.60 ± 2.09	97.58 ± 2.45	<0.001
Comorbidities			
Heart failure, *n* (%)	69 (28.63%)	72 (29.75%)	0.786
Atrial fibrillation, *n* (%)	64 (26.56%)	81 (33.47%)	0.097
CHD, *n* (%)	51 (21.16%)	60 (24.79%)	0.343
Laboratory parameters			
Ferritin	162.89 ± 105.57	1987.26 ± 5591.30	<0.001
Hemoglobin, g/dL	10.10 ± 2.36	9.40 ± 2.17	<0.001
WBC, 10^9^/L	10.31 ± 4.83	11.39 ± 10.07	0.328
Platelet, 10^9^/L	251.63 ± 169.95	190.04 ± 130.64	<0.001
Lymphocyte, %	15.83 ± 10.80	10.18 ± 10.50	<0.001
Creatinine, mg/dL	1.05 ± 0.79	1.85 ± 2.25	<0.001
Serum chloride, mg/dL	106.30 ± 6.57	105.12 ± 6.77	0.077
Anion gap, mg/dL	15.75 ± 3.55	17.51 ± 4.97	<0.001
Scoring systems			
GCS score	12.68 ± 3.29	12.44 ± 3.52	0.683
SOFA score	4.27 ± 3.20	6.30 ± 3.95	<0.001
OASIS score	31.47 ± 8.30	34.57 ± 8.76	<0.001
Clinical outcomes, *n* (%)			
30-day mortality	38 (15.77)	71 (29.34)	<0.001
90-day mortality	50 (20.75)	86 (35.54)	<0.001
One-year mortality	75 (31.12)	107 (44.21)	0.003
Length of stay in ICU	5.95 ± 7.33	8.94 ± 9.80	<0.001

SBP, Systolic blood pressure; DBP, Diastolic blood pressure; MAP, Mean arterial pressure; GCS, Glasgow Coma Scale; OASIS, Oxford acute severity of illness score; SOFA, Sequential organ failure assessment; ICU, Intensive Care Unit; WBC, White Blood Cell; CHD, Coronary heart disease; and BUN, Blood urea nitrogen.

### 3.2 Association of ferritin with 90-day, 30-day mortality, and 1-year mortality of cases with stroke

In our study, three models were constructed to assess independent prognostic roles of ferritin in stroke cases after adjustment of other possible confounders. 95% CI and effect sizes (HR) are listed in Table 2. Concerning 30-day mortality, the HR (95% CI) for a high Ferritin (≥373) was 1.925 (1.298, 2.854); *p* = 0.00113, compared to a low ferritin (< 373). After adjusting for age, sex, race, heart rate, SBP, DBP, hemoglobin, temperature, SPO2, SOFA, and GCS, the HR (95% CI) for a high Ferritin (≥373) was 1.782 (1.126, 2.820); *p* = 0.01367, compared to a low Ferritin (<373).

**Table 2:** HR (95% CIs) for all-cause mortality across groups of ferritin.

	Model 1^a^		Model 2^b^		Model 3^c^	
	HR (95% CIs)	*p* value	HR (95% CIs)	*p* value	HR (95% CIs)	*p* value
30-day all-cause mortality						
<373	1.0		1.0		1.0	
≥373	1.925(1.298, 2.854)	0.00113	2.138 (1.431, 3.195)	0.00021	1.782 (1.126, 2.820)	0.01367
90-day all-cause mortality						
<373	1.0		1.0		1.0	
≥373	1.829 (1.290, 2.592)	0.00069	2.030 (1.422, 2.898)	0.00010	1.870 (1.248, 2.804)	0.00244
1-year all-cause mortality						
<373	1.0		1.0		1.0	
≥373	1.569 (1.168, 2.109)	0.00280	1.787 (1.321, 2.417)	0.00016	1.641 (1.171, 2.300)	0.00244

Models 1, 2, and 3 were derived from Cox proportional hazards regression models.

^a^Model 1 covariates were adjusted for nothing.

^b^Model 2 covariates were adjusted for age, sex and race.

^c^Model 3 covariates were adjusted for age, sex, race, heart rate, SBP, DBP, hemoglobin, temperature, SPO2, SOFA, and GCS.

Using recursive algorithm and two-piecewise linear regression model, IP was calculated, which was 2,204. On the left side of the IP, there was a positive relationship between Ferritin and 30-day mortality, and the effect size, 95% CI and *p* value were 1.0006 (1.0004, 1.0009) *p* < 0.0001, respectively. On the right of the IP, the effect size, 95% CI and *p* value were 1.0000 (1.0000, 1.0000) and 0.3107, respectively.

### 3.3 Non-linear relationship

**Figure 1:**
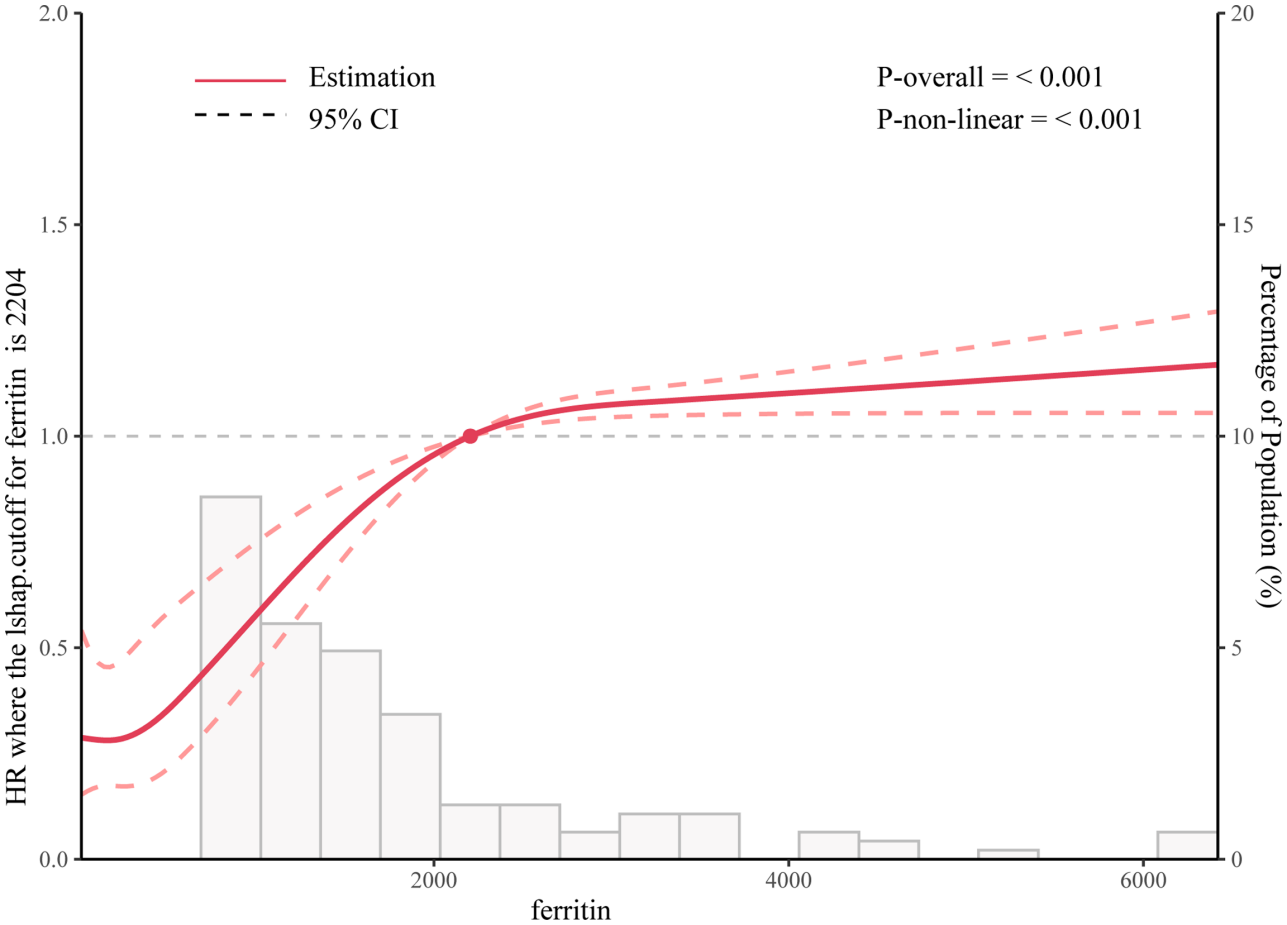
Association between ferritin levels and all-cause mortality.

Using the generalized additive model, non-linear relationship of ferritin with the 30-day mortality was analyzed (Figure 1). The two-piecewise linear regression model (LRM) and LRM were analyzed, and the *p* value was 0.012 revealed by the log-likelihood ratio test, demonstrating that the two-piecewise LRM fit the model. Using two-piecewise LRM and recursive algorithm, the IP was calculated, which was 2,204 (Table 3). On the left of the IP, there was a positive association of ferritin with 30-day mortality, and the effect size, 95%CI and *p* value were 1.0006 (1.0004, 1.0009), and < 0.0001, respectively. On the right side of the IP, the effect size, 95% CI, and *p* value were 1.0000 (1.0000, 1.0000) and 0.3107, respectively. L-shape between ferritin and 30-day mortality threshold of 2,204 was found. Taken together, it was demonstrated that ferritin had a threshold effect on the 30-day mortality.

**Table 3:** Nonlinearity addressing by weighted two-piecewise linear model.

Outcome	30-day mortality
	HR (95% CIs) *p* value
Linear regression model	1.0000 (1.0000, 1.0001) 0.0006
Two-piecewise linear regression model	
Inflection point	2,204
<2,204	1.0006 (1.0004, 1.0009) <0.0001
≥2,204	1.0000 (1.0000, 1.0000) 0.3107
Log likelihood ratio test	<0.001

## 4 Discussion

To our knowledge, this constitutes the first and most extensive comprehensive study that endeavors to probe the associations between ferritin levels and both short-term and long-term mortality in acute stroke patients. Our findings spotlight a significant relationship wherein elevated ferritin levels are intimately associated with an upsurge in all-cause mortality (encompassing both short-term and long-term contexts) in individuals navigating the perils of an acute stroke.

This study ventured into an exploration of the relationship between ferritin levels and all-cause mortality among stroke patients, revealing noteworthy associations and offering crucial insights into potential clinical ramifications and strategies. Our results hint that elevated ferritin levels might be concurrently linked with an augmented risk of all-cause mortality in these individuals, resonating with the plausible role of ferritin as not merely an iron storage marker but also a surrogate indicator of various pathophysiological processes.

The intimate liaison between ferritin levels and all-cause mortality in stroke patients unveils a compelling narrative that interweaves paths of iron metabolism, inflammation, and oxidative stress, subsequently exerting a substantial impact on neurological vulnerability and systemic homeostasis (17, 18). Ferritin, conventionally heralded for its role in iron storage, has progressively emerged as a biomarker that delineates intricate pathophysiological pathways and potentially foreshadows clinical outcomes in a myriad of conditions, including stroke (19, 20). Within the convoluted realms of stroke pathophysiology, the elevation of ferritin is not merely emblematic of altered iron homeostasis but, quite pivotally, serves as a beacon of systemic inflammatory responses (21). The perturbation in cerebral perfusion, resultant from a stroke, instigates a cascade of inflammatory mediators, thereby summoning peripheral immune cells to the neural locale. Ferritin levels surge as an acute-phase reactant, potentially mirroring the magnitude of this inflammatory maelstrom (22). This systemic inflammation, indelibly etched into the post-stroke biological response, not only augments neuronal injury but also potentially precipitates a series of systemic repercussions that might influence overall survival (23). Moreover, when dissecting the relationship between ferritin and oxidative stress, the pivotal role of iron, encapsulated within ferritin’s molecular embrace, comes to the forefront. Excess iron, particularly unbound or loosely bound iron, is a catalyst for the Fenton reaction, spawning reactive oxygen species that wreak oxidative havoc upon cellular structures, particularly within the neuraxis (24). The neural tissue, with its high metabolic demand and lipid-rich milieu, is particularly susceptible to oxidative injury, thereby exacerbating post-stroke neuronal death and potentially hindering neurological recovery (25, 26). In the vascular milieu, the potential atherogenicity of elevated ferritin or iron overload adds an additional layer of complexity to its relationship with stroke outcomes (27). By fostering a pro-oxidant environment, iron can facilitate the oxidation of low-density lipoproteins, a pivotal step in atherogenesis, thus potentially predisposing to subsequent vascular events, including recurrent strokes, which indubitably influence overall prognosis and mortality (28). Furthermore, the interpretational conundrum lies in discerning whether ferritin serves merely as a proxy for underlying inflammatory or oxidative processes or whether it directly modulates these pathways to influence post-stroke recovery and mortality. Therefore, while observational data might illuminate associations between ferritin levels and all-cause mortality, mechanistic insights are imperative to unravel the causal pathways, if any, and to discern whether ferritin modulation might serve as a therapeutic avenue in optimizing post-stroke care and outcomes (29, 30).

The observed non-linear relationship between Ferritin levels and 30-day mortality, where Ferritin acts as a risk factor when levels exceed 49.4 ng/mL and as a protective factor when below this threshold, can be attributed to several biological and clinical mechanisms. Ferritin, primarily known as an intracellular protein that stores iron, plays a crucial role in regulating iron homeostasis and protecting cells from iron-induced oxidative damage. At low Ferritin levels (<49.4 ng/mL), the protein’s ability to sequester and store iron mitigates the risk of free iron-catalyzed production of reactive oxygen species (ROS), thus preventing oxidative stress and cellular damage. This protective role is particularly important in critical illnesses, where oxidative stress can exacerbate tissue damage and inflammatory responses. Therefore, maintaining Ferritin within this lower range may enhance cellular resilience and improve survival outcomes. Conversely, elevated Ferritin levels (>49.4 ng/mL) are often indicative of acute or chronic inflammation, as Ferritin is an acute-phase reactant that increases in response to inflammatory stimuli. High Ferritin levels can reflect underlying conditions such as infections, liver disease, or malignancies, which are associated with poorer prognoses and higher mortality rates. Additionally, excessive Ferritin can contribute to iron overload, leading to increased ROS production and further oxidative stress, thereby exacerbating tissue injury and compromising physiological functions.

Beyond its conventional role as an iron-storage protein, ferritin has been implicated in several pathogenic pathways, especially those involving inflammation and oxidative stress. Pertinently in stroke contexts, these pathways are pivotal, as they can amplify neuronal injury and impede brain repair processes. Our findings, which suggest that elevated ferritin levels might symbolize an escalated inflammatory and oxidative state, thereby contributing to poorer post-stroke outcomes, align with this biological understanding (31). However, while some studies have highlighted associations between higher ferritin levels and adverse outcomes in varied populations (for instance, those with cardiovascular diseases or metabolic syndromes), others have presented ambiguous or conflicting findings. These inconsistencies highlight the intricate role of ferritin across disparate clinical scenarios and patient cohorts, potentially affected by multiple confounding factors such as comorbidities, age, and the acute phase of stroke. Hence, interpreting both our study’s results and those of others necessitates a mindful acknowledgment of ferritin’s multifaceted role and the varied clinical contexts.

Should further studies validate our findings, incorporating ferritin level assessments into prognostic models for stroke patients may potentially assist in risk stratification and guide therapeutic decision-making. For example, patients exhibiting elevated ferritin could be earmarked for more intensive monitoring or robust management, although the exact therapeutic implications warrant further exploration and confirmation via clinical trials.

It is paramount to recognize the limitations embedded in our study. Firstly, being a retrospective cohort study, it cannot establish a causal relationship between mortality and stroke. Secondly, although we endeavored to adjust for potential risk factors such as BMI, smoking status, and comorbidities, the presence of residual confounders, such as pro-inflammatory factors, marital status, and other known or unknown confounders, cannot be entirely negated. Thirdly, for ferritin, only measurements from the first 24 h of admission were selected, leaving the relationship between subsequent ferritin variations and prognosis unevaluated. The utilization of only baseline assessments heightens the risk of misclassification bias. Therefore, the outcomes and associations articulated should be interpreted with caution. Progressing ahead, mechanistic studies that delve into the causal pathways tying ferritin with suboptimal post-stroke outcomes are urgently needed. Moreover, future research should include prospective, multi-center studies to validate our findings and determine whether ferritin can be effectively leveraged as a prognostic biomarker in clinical practice.

## 5 Conclusion

In summation, this study sheds light on the potential association between ferritin levels and all-cause mortality in stroke patients, yet further research is critical to unveil the underpinning mechanisms and to authenticate these findings across varied settings and populations. With robust validation, the incorporation of ferritin into prognostic models may emerge as a clinically valuable tool to enhance patient care and management stroke.

## Data availability statement

The raw data supporting the conclusions of this article will be made available by the authors, without undue reservation.

## Ethics statement

The studies involving humans were approved by the Medical Information Mart for Intensive Care. The studies were conducted in accordance with the local legislation and institutional requirements. Written informed consent for participation in this study was provided by the participants’ legal guardians/next of kin.

## Author contributions

XX: Investigation, Software, Writing – original draft, Writing – review & editing. JL: Writing – review & editing, Project administration, Supervision, Visualization, Funding acquisition. WF: Data curation, Methodology, Supervision, Writing – original draft, Writing – review & editing. ZC: Conceptualization, Investigation, Software, Visualization, Writing – review & editing. JW: Writing – original draft, Writing – review & editing, Conceptualization, Investigation, Resources, Software, Visualization. HX: Funding acquisition, Resources, Visualization, Writing – original draft, Writing – review & editing.
